# Dose‐dependent effects of graded altered gravity during hypovolaemia on central haemodynamics and cardiovascular autonomic regulation

**DOI:** 10.1113/EP093840

**Published:** 2026-06-19

**Authors:** Adrien Robin, Cort D. Reinarz, Huc Pentinat‐Llurba, Syeda Yasmin Zaman, Christopher J. Rasmussen, Jason R. McKnight, Lisa Haddad, David C. Zawieja, Ana Diaz‐Artiles

**Affiliations:** ^1^ Aerospace and Extreme Environment Nursing Program, College of Nursing Texas A&M University Bryan Texas USA; ^2^ Department of Aerospace Engineering Texas A&M University College Station Texas USA; ^3^ Department of Mechanical and Aerospace Engineering University of California, Davis Davis California USA; ^4^ Exercise & Sport Nutrition Laboratory, Human Clinical Research Facility, Department of Kinesiology and Sports Management Texas A&M University College Station Texas USA; ^5^ Naresh K. Vashisht College of Medicine Texas A&M University College Station Texas USA; ^6^ Department of Medical Physiology Texas A&M University College Station Texas USA

**Keywords:** cardiovascular physiology, dose–response, extreme environment, microgravity, spaceflight, tilt table

## Abstract

The cardiovascular system is strongly influenced by gravitational loading and blood volume status. Quantifying central haemodynamic and autonomic reflex responses to fluid redistribution is important in altered‐gravity conditions, with relevance to spaceflight and clinical medicine. However, the continuous physiological response to graded gravitational loading under hypovolaemia has not been characterized. Here, we used graded tilt to derive continuous dose–response relationships to altered gravity during acute hypovolaemia. Sixteen healthy adults (8 males, 25 ± 2 years; 8 females, 23 ± 1 years) completed a graded tilt protocol from 45° head‐down to 45° head‐up in 15° increments, before and after oral furosemide administration (40 mg). Steady‐state haemodynamics and heart‐rate variability (HRV) were assessed at seated and supine baselines and at each tilt angle. Hypovolaemia (15.3 ± 0.2% reduction in plasma volume) increased heart rate and total peripheral resistance, while stroke volume and cardiac output decreased, with no significant change in arterial pressure. HRV decreased in the time domain and shifted towards sympathetic predominance in the frequency domain. Continuous dose–response curves were generated by projecting the gravitational vector along the cranio‐caudal axis across tilt, enabling inclusion of Moon (∼0.17*G* at 9° head‐up) and Mars (∼0.38*G*, at 22° head‐up) equivalent gravity levels within the model. These findings provide an integrative continuous cardiovascular dose–response framework for altered gravity under hypovolaemia. This framework may help guide development of countermeasures across specific gravitational loads and inform conditions in which orthostatic stress and impaired volume regulation coexist.

## Abstract (highlights)

- **What is the central question of this study?**
  How does acute hypovolaemia modify the central haemodynamic and cardiovascular autonomic dose–response to graded gravitational stress across both head‐down and head‐up tilt?
- **What is the main finding and its importance?**
  Using a head‐down and a head‐up tilt paradigm, we generated gravitational dose–response curves to assess central haemodynamic and cardiovascular autonomic response to acute hypovolaemia. Our modelled curves include Moon and Mars equivalent gravity levels, informing the development of spaceflight countermeasures adapted to specific gravitational loads or Earth‐based clinical contexts.

## 1 INTRODUCTION

A defining early physiological effect of microgravity during spaceflight is a headward redistribution of blood and fluid driven by the removal of the hydrostatic pressure gradient along the body axis (Norsk, 2020), (Moore & Thornton, 1987; Thornton et al., 1987). This fluid shift contributes to facial oedema and reduced leg volume and initiates broader cardiovascular and autonomic adaptations, including reduced plasma volume (PV), altered vascular resistance and compliance, changes in baroreflex function, and cardiac remodelling. During spaceflight and ground‐based altered‐gravity, autonomic cardiovascular control is altered, including changes in baroreflex responses, which may contribute to orthostatic intolerance (Jordan et al., 2022). On Earth, daily transitions between upright and supine postures provide recurring gravitational challenges that help maintain cardiovascular reflex control; in microgravity, the absence of these orthostatic stressors contributes to cardiovascular deconditioning and orthostatic intolerance upon return to 1*G*.

Future exploration missions involving transitions between gravitational environments (e.g., partial gravity and 1*G*) motivate a quantitative understanding of how different ‘doses’ of gravitational stress shape haemodynamic and autonomic responses, which is essential for countermeasure optimization. Proposed countermeasures include lower‐body negative pressure (Hall et al., 2024; Whittle et al., 2025) and artificial gravity (Diaz‐Artiles et al., 2018; Diaz‐Artiles, Heldt et al., 2019), but their effectiveness likely depends on selecting appropriate gravitational doses for a given individual and mission phase.

To reproduce an altered gravity environment, head‐down bedrest (Robin, Wang et al., 2022), dry‐immersion (Horeau et al., 2024; Robin et al., 2023; Robin, Navasiolava et al., 2022; Twomey et al., 2021) and parabolic flight (Pentinat‐Llurba et al., 2026) are used as human models. Tilt table protocols provide a complementary framework by systematically varying body orientation relative to the gravity vector, thereby modulating the body‐axis gravitational component (*G_z_*) in a controlled and quantifiable manner. While recent studies have characterized responses across multiple tilt angles (Petersen et al., 2022; Whittle & Diaz‐Artiles, 2023; Whittle et al., 2022), they have largely focused on healthy participants, limiting extrapolation to deconditioned individuals. Thus, a dose–response characterization under acute hypovolaemia, a deconditioning component observed during microgravity exposure and a frequent Earth‐based contributor to orthostatic intolerance, is needed to guide countermeasure design and inform clinically relevant postural challenges.

The aim of this study was to construct cardiovascular dose–response curves describing central haemodynamic and autonomic responses across a wide range of head‐down and head‐up tilt angles before and during hypovolaemia. Although dose–response relationships between gravitational loading and cardiovascular responses have been characterized under normovolaemic conditions, comparable characterizations of the continuous cardiovascular response across gravitational loading during hypovolaemia are lacking. By mapping experimental data across multiple gravitational levels rather than at a single angle, the resulting dose–response models capture the continuous physiological response within a representative population. We hypothesized that acute hypovolaemia would shift these dose–response relationships toward greater cardiovascular and autonomic strain, characterized by lower stroke volume (SV) and cardiac output (CO), higher heart rate and peripheral resistance, and reduced vagal modulation, particularly at higher head‐up tilt angles. Our work thus informs countermeasure dosing across gravitational transitions where hypovolaemia and postural stress co‐occur.

## 2 METHODS

### 2.1 Ethical approval

All participants received verbal and written explanations of the study protocol and provided written informed consent. The study complied with the *Declaration of Helsinki* (1964, and its later amendments) and was approved by the Texas A&M Human Research Protection Program (IRB No. STUDY2023‐0049).

### 2.2 Participants

Seventeen healthy, recreationally active adults from the Texas A&M University System were recruited. One male participant experienced a brief orthostatic intolerance episode while seated before the tilt manoeuvre during the hypovolaemia protocol; he quickly fully recovered and his test was terminated and data excluded. Thus, 16 participants (8 males, 8 females) were included in the final analysis. One of these male participants completed the hypovolaemia session but developed presyncope symptoms (light‐headedness, sudden heart rate increase) at the final tilt angle of 45° head up tilt (HUT); the protocol was stopped, he was returned to a horizontal position, and symptoms resolved without sequelae. All other participants completed the full protocol without adverse events.

To minimize potential confounders, the age range of selected subjects was restricted to young adults. Before participating, subjects completed a screening questionnaire to identify any exclusion criteria, including current use of any cardiac, blood pressure, muscle relaxant, anticoagulant or stimulant medications, thyroid disease, cardiovascular pathologies, extreme obesity, and history of hypertension. Female participants were tested during the follicular phase of the menstrual cycle, and those using combined oral oestrogen–progesterone contraceptives were excluded to reduce hormonal influences. Participant characteristics (mean ± SE), including screening blood pressure, are provided in Table 1.

**TABLE 1:** Baseline characteristics of the 16 recreationally active participants.

Characteristic	Value (mean ± SE)			*P* (males versus females)
	Total (*n* = 16)	Males (*n* = 8)	Females (*n* = 8)	
Age (years)	24.4 ± 1.0	25.4 ± 1.8	23.4 ± 0.8	0.345
Height (cm)	168.4 ± 2.1	174.5 ± 1.8	162.4 ± 2.4	**<0.001**
Weight (kg)	67.4 ± 3.0	77.2 ± 2.5	57.5 ± 2.1	**0.001**
BMI (kg m^−2^)	23.6 ± 0.8	25.5 ± 1.1	21.8 ± 0.5	**0.014**
MAP (mmHg)	98.9 ± 3.5	105.7 ± 5.3	93.0 ± 3.8	0.078
SBP (mmHg)	120.4 ± 3.5	122.2 ± 6.3	118.9 ± 3.9	0.668
DBP (mmHg)	78.5 ± 3.0	84.6 ± 4.3	73.2 ± 3.4	0.060

*Note*: Data are presented as mean ± SE. Data recorded during the seated baseline before any tilt intervention. Male–female comparisons used two‐sided Welch's *t*‐test. *P*‐values in bold indicate statistical significance. Abbreviations: BMI, body mass index; DBP, diastolic blood pressure; MAP, mean arterial blood pressure; SBP, systolic blood pressure.

### 2.3 Experimental design and testing protocol

**FIGURE 1:**
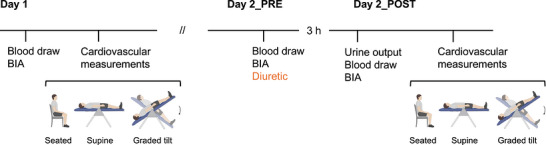
Protocol configuration and deconditioning. Deconditioning was induced by acute hypovolaemia using oral furosemide (40 mg). Blood draws and bioimpedance (BIA) were performed to assess hydration status and PV changes. Cardiovascular measurements consisted in central haemodynamic and autonomic data collection during the tilt protocol. The tilt protocol consisted in a seated then supine baseline, followed by a graded tilt test from 45° head down (HDT) to 45° head up (HUT) by 15° increment. BIA: bioimpedance. Created with BioRender.com (full version).

Each participant completed two experimental sessions on separate days. The mean interval between the two experimental sessions was 1.9 ± 0.3 days (SE) with a maximum of 4 days between sessions (as depicted in Figure 1). Participants were instructed to avoid caffeine intake and physical exercise prior to each session. Sleep and dietary intake were not formally standardized or recorded. Participants were instructed to maintain their usual morning eating habits (or consume at least a minimal breakfast). For one participant, testing was postponed because of insufficient sleep. On Day 1 (control condition), participants arrived at the clinical facility at 08.30 h. Upon arrival, venous blood samples, anthropometric measurements and bioimpedance assessments were obtained. At 09.00 h, participants performed baseline data collection and a graded tilt test ranging from 45° head‐down tilt (HDT) to 45° head‐up tilt (HUT) in 15° increments. On Day 2 (hypovolaemia condition), the initial procedures were identical to those on Day 1 until 09.00 h. After confirming systolic blood pressure to be >100 mmHg, participants ingested oral furosemide (40 mg) to induce hypovolaemia via PV reduction. To prevent hypokalaemia, an oral potassium supplement (40 mEq) was administered concurrently. Blood pressure was monitored every 30 min using an automated arm cuff throughout the subsequent 3‐h waiting period. This waiting period allowed for diuresis and a measurable reduction in PV to occur before tilt testing. At approximately 12.00 h, following the 3‐h diuretic period, participants began the post‐diuretic baseline data collection and graded tilt protocol. This second testing session lasted approximately 2.5 h.

Within each session (control or hypovolaemia), data collection followed a standardized sequence: seated baseline, supine baseline (horizontal), and the graded tilt protocol using a tilt table (World Triathlon, Tampa Bay, FL, USA) at seven angles (45° HDT, 30° HDT, 15° HDT, 0° [horizontal], 15° HUT, 30° HUT and 45° HUT).

At each measurement point (seated baseline, supine baseline and each tilt angle), participants rested quietly for 5 min to allow haemodynamic stabilization. Following this rest period, discrete measurements of CO and related parameters were obtained using an inert gas rebreathing system (Innocor, Cosmed, Rome, Italy). Throughout the entire protocol, continuous recordings of blood pressure and electrocardiography (ECG) were collected. Each measurement block (rest period plus data collection) lasted approximately 15 min.

### 2.4 Hydration status

Following furosemide administration, urine output was measured using a graduated urine collection container during the 3‐h waiting period to quantify diuresis. Venous blood samples were collected on Day 1 before the tilt session (control), and on Day 2 before and after the 3‐h diuretic period (deconditioning). Changes in PV were estimated using the Dill and Costill method (Dill & Costill, 1974). Haemoglobin (Hb) and haematocrit (Hct) values were used to calculate the percentage change in PV relative to baseline (i.e., prior to diuretic ingestion):

(1) $$\begin{array}{ccc} \Delta \mathrm{PV}\left(\%\right) & = & \left(\left[\mathrm{HbB}\right]\times \left(1-0.01\left[\mathrm{Hcti}\right]\right)\right)/\left(\left[\mathrm{Hbi}\right]\times \left(1-0.01\left[\mathrm{HctB}\right]\right)\right)\\ & & \times \;100-100 \end{array}$$

where HbB and HctB represent baseline haemoglobin and haematocrit levels, respectively, and Hbi and Hcti correspond to haemoglobin and haematocrit values at subsequent time points.

Body composition was assessed via bioimpedance analysis (BIA) using a Bodystat QuadScan 4000 device (Bodystat Ltd, Sulby, Isle of Man), performed in the supine position immediately following blood collection. Whole‐body wrist‐to‐ankle measurement were used to estimate total body water (TBW), extracellular water (ECW) and intracellular water (ICW).

### 2.5 Central haemodynamic measurements

Haemodynamic variables were collected as described previously in Whittle et al. (2022). Briefly, SV and CO were measured using an Innocor inert gas rebreathing system (Cosmed). Oxygen consumption (${\dot{V}}_{O_{2}}$) was also retained as a secondary variable, as it was obtained concurrently with CO during gas rebreathing and provided complementary functional context for interpretation of the haemodynamic responses. Continuous beat‐to‐beat arterial blood pressure, heart rate and derived autonomic indices were obtained using a Finapres NOVA device (Finapres Medical Systems B.V., Enschede, the Netherlands). Finapres data were recorded continuously throughout the experimental protocol. Following visual inspection of all traces, values were averaged over the sixth minute at each tilt angle to obtain a single representative value for each condition and participant. Pressure readings were corrected to heart level using a hydrostatic height sensor and were calibrated at each measurement point with a brachial blood pressure reference.

### 2.6 Autonomic nervous system measurements

Autonomic indices were also obtained using a Finapres NOVA (Finapres Medical Systems). Autonomic analysis was based on heart rate variability (HRV) and baroreflex sensitivity (BRS). Time‐domain measures included the standard deviation of NN intervals (SDNN), the root mean square of successive differences of NN interval (RMSSD), and the HRV triangular index (HRVTi). BRS was also included as a time‐dependent measure of autonomic function. Frequency‐domain measures included the spectral power density in the low‐frequency (LF, 0.04–0.15 Hz) and high‐frequency (HF, 0.15–0.4 Hz) bands, expressed in both absolute and normalized units (LFNorm and HFNorm), as well as the LF/HF ratio. Three of the four time‐domain measures (SDNN, RMSSD and HRVTi) and all the frequency‐domain measures were continuously calculated using a 300‐s sliding window. BRS was derived using the built‐in cross‐correlation xBRS approach (Westerhof et al., 2004) based on the spontaneous relationship beat‐to‐beat SBP and RR interval, resampled at 1 Hz, consistent with the method described by Westerhof et al.

### 2.7 Statistical analysis

Data are presented as means ± SE for bar and line graphs, and in the text. In box plots, boxes indicate 25^th^ percentile, median and 75^th^ percentile, and whiskers indicate 1.5 × interquartile range (IQR) values.

For hydration‐related variables, linear mixed‐effects models (LMMs) were fit with Day as a fixed effect (Day 1, Day 2_PRE, Day 2_POST) and a random intercept for Subject to account for repeated measures, with Day 2_PRE used as the reference. The global effect was tested on the fitted model, and *post hoc* comparisons vs. Day 2_PRE were performed using Dunnett‐adjusted contrasts.

Central haemodynamic and autonomic outcomes were analysed using LMMs or generalized linear mixed‐effects models (GLMMs; gamma distribution with log link), depending on outcome distribution. Models included fixed effects for *Volaemia* (Control vs. Hypovolaemia), *Angle*, and their interaction (*Volaemia* × *Angle*), with a random intercept for Subject to account for repeated measures. Type III tests of fixed effects were computed and following *post hoc* contrasts were derived from estimated marginal means (emmeans) to compare Control vs. Hypovolaemia at each *Angle* (false discovery rate FDR adjustment) and to compare each *Angle* to the supine baseline within each *Volaemia* condition (Dunnett adjustment). Model assumptions were assessed visually using residual diagnostic panels for LMMs and simulation‐based scaled residual diagnostics for all models (DHARMa), with DHARMa used as the primary diagnostic for GLMMs. To assess sex‐related effects, secondary analyses were performed by adding *Sex* as a fixed factor to the mixed‐effects models, including its interactions with *Volaemia* and *Angle*. For each variable, missing values were not included, and mixed‐effects models were fitted to the remaining repeated observations without imputation.

To complement the primary mixed‐effects analyses, paired standardized effect sizes were calculated for each haemodynamic and autonomic outcome. Values were first averaged within subject and within condition across the graded tilt angles, to have one summary value per subject for each condition. Paired Cohen's *d_z_* was then computed as the mean within‐subject difference divided by the standard deviation of the within‐subject differences.

Gravitational dose–response curves from 45° HDT to 45° HUT were constructed by refitting mixed‐effects models using a sine‐transformed tilt angle as the gravitational dose. Seated and supine baseline angles were excluded, following previously described methods (Whittle et al., 2022). For most outcomes, dose–response models were fit as LMMs (Gaussian) or gamma GLMMs (log link), whereas HR and SV were modelled using generalized additive mixed‐effects models (GAMMs; mgcv) to accommodate non‐linearity, allowing condition‐specific smooths when supported by model comparison. Curves are presented as model‐predicted means with 95% confidence intervals. We projected the gravitational vector along the cranio‐caudal axis for tilt as: *G* level: *G_z_* = 1 × sin(θ), allowing Moon and Mars gravity equivalent levels to be displayed (∼0.17*G* at ∼9° HUT and ∼0.38*G* at ∼22° HUT, respectively).

Statistical analyses were performed in R (version 4.4.1; RStudio 2024.09). LMMs were fit using *lme4* and GLMMs using *glmmTMB*; GAMMs were fit using *mgcv*. Adjusted means and contrasts were obtained with *emmeans*. Model diagnostics were evaluated visually using *ggResidpanel* for LMM residual plots and DHARMa simulation‐based scaled residual diagnostics (primary diagnostic for GLMMs). Significance was set at α = 0.05 (two‐sided).

## 3 RESULTS

### 3.1 Hydration status

To induce deconditioning, we used furosemide as an oral diuretic (40 mg) and performed the second tilt 3 h after ingestion, as depicted in Figure 1. Table 2 summarizes the changes in volaemia and body water compartments. Diuresis over the 3‐h period (i.e., from Day 2 PRE to Day 2 POST) resulted in a urine output of 1881 ± 103 mL. This was accompanied by significant increases in haemoglobin (*P* < 0.001) and haematocrit (*P* < 0.001) from Day 2 PRE to Day 2 POST, and a significant decrease in PV of about 15.3 ± 1.5% (*P* < 0.001), reflecting the induced hypovolaemia on Day 2 POST compared to Day 2 PRE.

**TABLE 2:** Volaemia status and changes in body water compartment.

Outcome		Day 1	Day 2 PRE	Day 2 POST
Urine output (mL)		—	—	1881 ± 103
Haemoglobin (g dL^−1^)		14.5 ± 0.5	14.2 ± 0.4	15.5 ± 0.5^*^
Haematocrit (%)		43.8 ± 1.3^*^	42.6 ± 1.1	46.9 ± 1.3^*^
Plasma volume (Δ%)		−4.2 ± 1.5^*^	—	−15.3 ± 1.5^*^
Total body water (L)	Raw value	37.7 ± 2.1	37.7 ± 2.0	36.3 ± 1.9^*^
	Δ% vs. Day 2 PRE	−0.1 ± 0.6	—	−3.9 ± 0.3^*^
Extracellular water (L)	Raw value	16.4 ± 0.7	16.4 ± 0.6	15.6 ± 0.6^*^
	Δ% vs. Day 2 PRE	−0.3 ± 0.6	—	−4.8 ± 0.3^*^
Intracellular water (L)	Raw value	21.0 ± 1.5	21.0 ± 1.4	20.5 ± 1.4^*^
	Δ% vs. Day 2 PRE	−0.1 ± 0.5	—	−2.4 ± 0.3^*^

*Note*: Urine output was quantified during the 3‐h period following ingestion of furosemide (40 mg). Haemoglobin (Hb, g/dL) and haematocrit (Hct, %) were measured from venous blood samples on Day 1, and on Day 2 before and 3 h after diuretic administration. Changes in plasma volume (PV) were estimated using the Dill and Costill method (ΔPV (%) = ([HbB] × (1 – 0.01[Hcti]))/([Hbi] × (1 – 0.01[HctB])) × 100 – 100, where HbB and HctB represent baseline values, and Hbi and Hcti are values at subsequent time points). Body water compartments variations were estimated by whole‐body bioimpedance. ^*^Statistically significant differences (*P* < 0.05) compared to Day 2 PRE. Data are presented as mean ± SE.

Whole‐body BIA was used to assess hydration status and quantify TBW, ECW and ICW. The diuretic protocol resulted in a significant overall decrease in body water in all participants on Day 2 POST compared to Day 2 PRE. Specifically, TBW decreased from 37.7 ± 2.0 L to 36.3 ± 1.9 L (∆ TBW = −3.9 ± 0.3%, *P* < 0.001), ECW decreased from 16.4 ± 0.6 L to 15.6 ± 0.6 L (∆ ECW = −4.8 ± 0.3%, *P* < 0.001), and ICW decreased from 21.0 L ± 1.4 to 20.5 ± 1.4 L (∆ ICW = −2.4 ± 0.3%, *P* < 0.001).

We further examined the relationship between changes in body water and urine output during diuresis. Body water loss was significantly correlated with the urine volume (TBW: *r* = −0.757, *P* = 0.003; ECW: *r* = −0.714, *P* = 0.004; ICW: *r* = −0.568, *P* = 0.043), indicating that a greater diuresis was associated with a larger reduction in body water.

### 3.2 Central haemodynamic response

**FIGURE 2:**
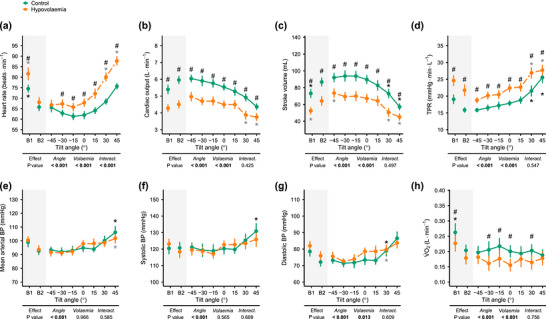
Hypovolaemia effect on central haemodynamic variables as a function of tilt angle. (a) Heart rate; (b) cardiac output; (c) stroke volume; (d) TPR, total peripheral resistance; (e) mean arterial blood pressure; (f) systolic blood pressure (BP); (g) diastolic blood pressure (BP); (h) ${\dot{V}}_{O_{2}}$, oxygen consumption. Measurements were collected at a seated baseline (B1), supine baseline (B2), 45° head‐down tilt (HDT), 30° HDT, 15° HDT, 0°, 15° head‐up tilt (HUT), 30° HUT and 45° HUT. *Angle*, *Volaemia* and *Interaction* effects were assessed with linear or generalized linear mixed‐effects models as described in the main text. Data (*n* = 16) are presented as means ± SE at each *Angle*. Asterisks (*; black, control; grey, hypovolaemia) indicate statistically significant (*P* < 0.05) differences between a specific *Angle* condition and the supine baseline condition B2 (*Angle* effect). #Statistically significant (*P* < 0.05) differences between control and hypovolaemia interventions at a given angle (*Volaemia* effect). B1, seated baseline; B2, supine baseline.

Changes in central haemodynamic variables across tilt angles, before and during hypovolaemia, are shown in Figure 2 (means ± SE), including the seated (B1) and supine (B2) baselines. All haemodynamic variables showed a significant *Angle* effect, and all variables except mean, systolic and diastolic blood pressure (MAP, SBP, DBP, respectively) showed a significant *Volaemia* effect. Sex effects are reported in Table 3; the *Sex* × *Volaemia* interaction was not significant.

**TABLE 3:** Statistical results of the linear mixed model and generalized linear mixed model analysis incorporating sex differences.

	*P*			
Variable	Global sex effect	*Sex* × *Angle*	*Sex* × *Volaemia*	*Sex* × *Angle* × *Volaemia*
Haemodynamic measurements				
HR	0.204	0.224	0.538	0.842
SV	0.033^*^	0.048^*^	0.445	0.077
CO	0.024^*^	0.728	0.594	0.458
TPR	0.535	0.704	0.362	0.601
SBP	0.002^*^	0.098	0.805	0.330
DBP	0.082	0.262	0.471	0.907
MAP	0.043^*^	0.148	0.971	0.575
${\dot{V}}_{O_{2}}$	<0.001^*^	0.652	0.917	0.629
Time‐domain autonomic indices				
SDNN	0.259	0.506	0.600	0.148
RMSSD	0.302	0.223	0.500	0.925
HRVTi	0.192	0.536	0.037^*^	0.658
BRS	0.437	0.223	0.423	0.580
Frequency‐domain autonomic indices				
LF	0.042^*^	0.267	0.534	0.546
HF	0.398	0.045^*^	0.184	0.516
LFNorm	0.515	0.020^*^	0.588	0.205
HFNorm	0.354	0.020^*^	0.138	0.163
LF/HF	0.314	0.108	0.652	0.707

* Statistically significant (*P* < 0.05) differences. See text for abbreviations and model details.

Heart rate (HR) increased with progressive tilt toward HUT and was higher during hypovolaemia (Figure 2a). At B1, HR was 7.1 ± 2.1 beats min^−^^1^ higher in hypovolaemia than in control (+9.5 ± 3.0%). Across all tilt conditions, HR remained higher in hypovolaemia by 6.8 ± 1.1 beats min^−^^1^ (+10.3 ± 1.7%, *d_z_* = 1.61), indicating resting tachycardia during hypovolaemia.

CO and SV decreased with tilt toward HUT and were lower during hypovolaemia (Figure 2b,c). At B1, CO was 1.1 ± 0.3 L min^−^^1^ lower in hypovolaemia than in control (−18.8 ± 5.7%), and the difference persisted across tilts (−1.0 ± 0.2 L min^−^^1^, −17.6 ± 2.9%, *d_z_* = −1.52). Similarly, SV was lower in hypovolaemia at B1 (−20.5 ± 3.7 mL, −27.2 ± 5.0%) and across tilts (−20.1 ± 3.0 mL, −24.1 ± 3.1%, *d_z_* = −1.77).

Total peripheral resistance (TPR) increased with tilt toward HUT and was higher during hypovolaemia (Figure 2d). At B1, TPR was 5.6 ± 2.1 mmHg min L^−^^1^ higher in hypovolaemia than in control (+36.3 ± 12.7%), and remained higher across tilts (+3.7 ± 0.9 mmHg min L^−^^1^, +20.8 ± 5.2%, *d_z_* = 1.15).

Blood pressure variables increased with tilt toward HUT, with no significant differences between control and hypovolaemia. MAP was higher than B2 only at 45° HUT in both conditions (Figure 2e), with an effect size *d_z_* = −0.004. SBP was higher than B2 only at 45° HUT in the control condition (Figure 2f), with an effect size *d_z_* = −0.14. DBP was higher than B2 only at 45° HUT in both conditions (Figure 2 g), with an effect size *d_z_* = 0.37.

Finally, oxygen consumption (${\dot{V}}_{O_{2}}$) was lower during hypovolaemia than in control at B1 (−8.1 ± 14.1%) and across tilts (−16.5 ± 4.7%) (Figure 2h), with an effect size *d_z_* = −0.83.

### 3.3 Time‐domain autonomic system response

**FIGURE 3:**
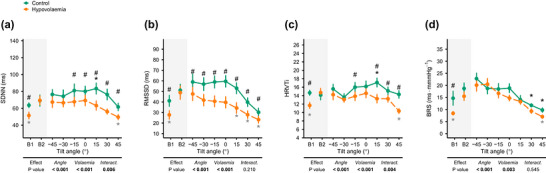
Hypovolaemia effect on time‐domain autonomic indices as a function of tilt angle. (a) SDNN, standard deviation of NN intervals (normalized RR intervals); (b) RMSSD, root mean square of successive differences; (c) HRVTi, heart rate variability triangular index; (d) BRS, baroreflex sensitivity using built‐in cross‐correlation approach (xBRS). Measurements were collected at a seated baseline (B1), supine baseline (B2), 45° head‐down tilt (HDT), 30° HDT, 15° HDT, 0°, 15° head‐up tilt (HUT), 30° HUT and 45° HUT. *Angle*, *Volaemia* and *Interaction* effects were assessed with linear or generalized linear mixed‐effects models as described in the main text. Asterisks (*; black, control; grey, hypovolaemia) indicate statistically significant (*P* < 0.05) differences between a specific *Angle* condition and the supine baseline condition B2 (*Angle* effect). #Statistically significant (*P* < 0.05) differences between control and hypovolaemia interventions at a given angle (*Volaemia* effect). Data (*n* = 16) are presented as means ± SE at each *Angle*. B1: seated baseline, B2: supine baseline.

Changes in time‐domain autonomic indices across tilt angles, before and during hypovolaemia, are shown in Figure 3 (means ± SE), including the seated (B1) and supine (B2) baselines. All time‐domain indices exhibited significant *Angle* and *Volaemia* effects. Sex effects are reported in Table 3; the *Sex* × *Volaemia* interaction was not significant, except for HRVTi.

SDNN decreased with progressive tilt toward HUT and was lower during hypovolaemia (Figure 3a). At B1, SDNN was 13.4 ± 3.8 ms lower in hypovolaemia than in control (−20.9 ± 6.0%), and remained lower across tilts (−11.1 ± 3.1 ms, −14.2 ± 4.2%, *d_z_* = −0.98).

RMSSD similarly decreased with tilt and was lower during hypovolaemia (Figure 3b). At B1, RMSSD was 14.1 ± 6.4 ms lower (−27.5 ± 8.4%), and remained lower across tilts (−13.1 ± 3.6 ms, −24.8 ± 5.5%, *d_z_* = −1.00).

HRVTi decreased with tilt and was lower during hypovolaemia (Figure 3c). At B1, HRVTi was 3.3 ± 0.9 lower (−21.3 ± 5.9%), and across tilts it was 1.9 ± 0.6 lower (−11.8 ± 4.3%, *d_z_* = −0.82), with the most pronounced reduction at 45° HUT.

BRS decreased with progressive tilt toward HUT, without significant differences at any specific tilt angle except for B1 (Figure 3d). At B1, BRS was 6.6 ± 3.3 ms mmHg^−^^1^ lower in hypovolaemia than in control (−24.7 ± 11.8%, *d_z_* = −0.41), whereas no significant hypovolaemia–control differences were observed across tilt angles.

### 3.4 Frequency‐domain autonomic system response

**FIGURE 4:**
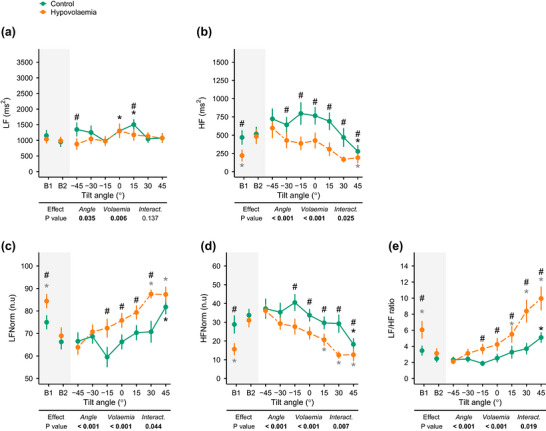
Hypovolemia effect on frequency‐domain autonomic indices as a function of tilt angle. (a) LF, power density in the low frequency range (0.04–0.15 Hz); (b) HF, power density in the high frequency range (0.15–0.4 Hz); (c) LFNorm, LF in normalized units (n.u.); (d) HFNorm, HF in normalized units (n.u.); (e) LF/HF ratio, ratio of low to high power densities. Measurements were collected at a seated baseline (B1), supine baseline (B2), 45° head‐down tilt (HDT), 30° HDT, 15° HDT, 0°, 15° head‐up tilt (HUT), 30° HUT and 45° HUT. *Angle*, *Volaemia* and *Interaction* effects were assessed with linear or generalized linear mixed‐effects models as described in the main text. Asterisks (*; black, control; grey, hypovolemia) indicate statistically significant (*P* < 0.05) differences between a specific *Angle* condition and the supine baseline condition B2 (*Angle* effect). #Statistically significant (*P* < 0.05) differences between control and hypovolemia interventions at a given angle (*Volaemia* effect). Data (*n* = 16) are presented as means ± SE at each *Angle*. B1: seated baseline, B2: supine baseline.

Changes in frequency‐domain autonomic indices across tilt angles, before and during hypovolaemia, are shown in Figure 4 (means ± SE), including the seated (B1) and supine (B2) baselines. All frequency‐domain indices exhibited a significant *Angle* effect and a significant *Volaemia* effect. Sex effects are reported in Table 3; the *Sex* × *Volaemia* interaction was not significant.

LF power increased during tilt compared with B2 (Figure 4a), with the most evident increases in the control condition around 0° and 15° HUT, with an effect size *d_z_* = −0.52. HF power decreased with progressive tilt toward HUT and was lower during hypovolaemia (Figure 4b). At B1, HF was 251.7 ± 112.3 ms^2^ lower in hypovolaemia than in control (−45.1 ± 11.4%), and remained lower across tilts (−254.8 ± 69.9 ms^2^, −37.2 ± 8.8%, *d_z_* = −0.98).

The normalized LF (LFNorm: LF power divided by total power) significantly increased with increasing tilt toward HUT, being higher during hypovolaemia (Figure 4c). During hypovolaemia, in seated baseline, LFNorm was 7.9 ± 4.1 higher (+13.4 ± 7.9%) and was 7.3 ± 2.4 higher across tilt (+12.3 ± 4.3%, *d_z_* = 0.85). The normalized HF (HFNorm: HF power divided by total power) significantly decreased with increasing tilt toward HUT, being lower during hypovolaemia (Figure 4d). During hypovolaemia, in seated baseline, HFNorm was 13.0 ± 6.3 lower (−33.8 ± 12.2%) and was 8.2 ± 3.1 lower across tilt (−20.7 ± 6.2%, *d_z_* = −0.73).

Finally, LF/HF ratio significantly increased with increasing tilt toward HUT, being higher during hypovolaemia (Figure 4e). During hypovolaemia, in seated baseline, the LF/HF ratio was 2.0 ± 1.0 higher (+107.7 ± 64.7%) and was 2.2 ± 0.5 higher across tilt (+92.6 ± 27.8%, *d_z_* = 1.30).

### 3.5 Dose–response curves

**FIGURE 5:**
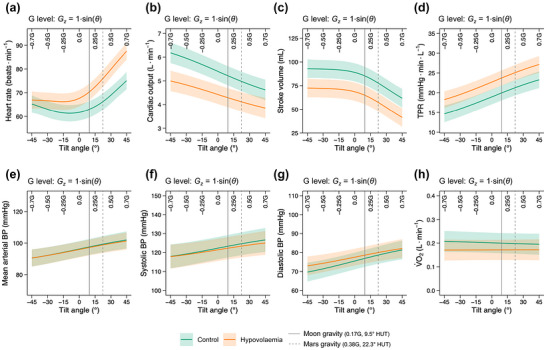
Estimated gravitational dose–response curves for haemodynamic parameters as a function of tilt angle. (a) Heart rate; (b) cardiac output; (c) stroke volume; (d) TPR, total peripheral resistance; (e) mean arterial blood pressure; (f) systolic blood pressure (BP); (g) diastolic blood pressure (BP); (h) ${\dot{V}}_{O_{2}}$, oxygen consumption. Curves were fitted from experimental data with LMMs (CO, TPR, MAP, SBP, DBP, ${\dot{V}}_{O_{2}}$) or GAMMs (HR, SV) models as described in the main text. Curves are presented as means ± 95% confidence interval. The gravitational vector is projected along the cranio‐caudal axis for tilt as: *G* level: *G_z_* ​ = 1 × sin(θ). The vertical lines indicate the approximate partial gravity levels of the Moon (continuous line) and Mars (dashed line).

**FIGURE 6:**
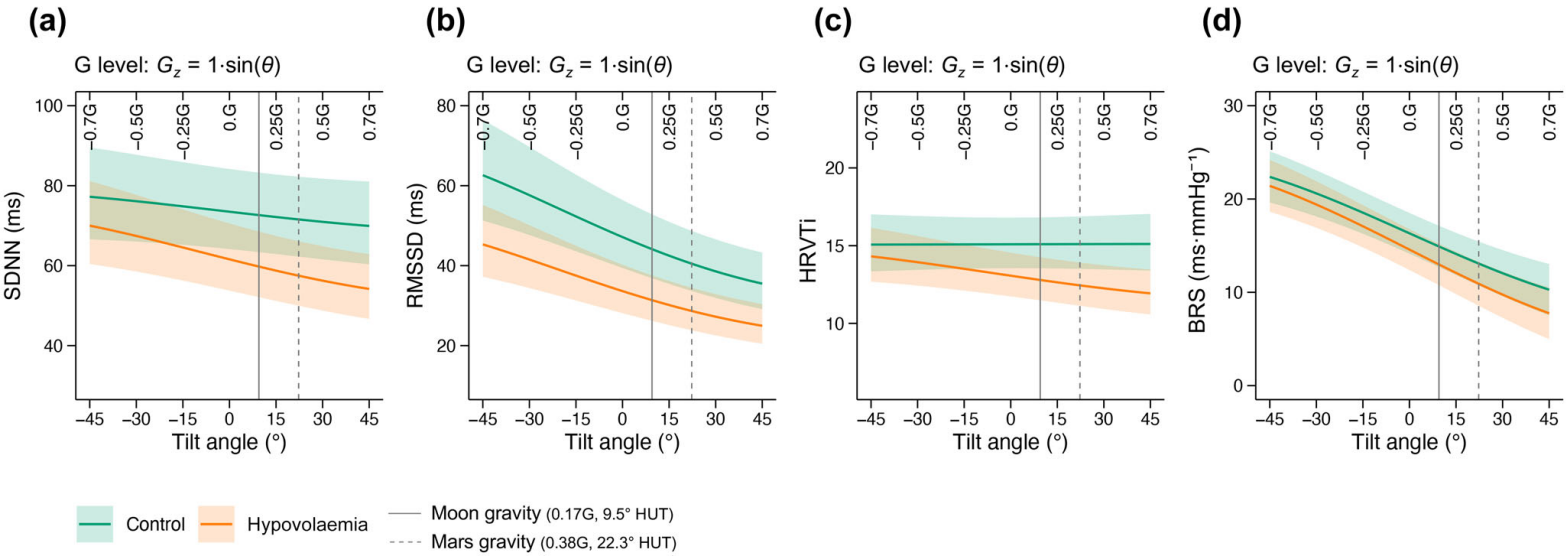
Estimated gravitational dose–response curves for time‐domain autonomic indices as a function of tilt angle. (a) SDNN, standard deviation of NN intervals (normalized RR intervals); (b) RMSSD, root mean square of successive differences; (c) HRVTi, heart rate variability triangular index; (d) BRS, baroreflex sensitivity using built‐in cross‐correlation approach (xBRS). Curves were fitted from experimental data with LMMs or GLMMs models as described in the main text. Curves are presented as means ± 95% confidence interval. The gravitational vector is projected along the cranio‐caudal axis for tilt as: *G* level: *G_z_* ​ = 1 × sin(θ). The vertical lines indicate the approximate partial gravity levels of the Moon (continuous line) and Mars (dashed line).

**FIGURE 7:**
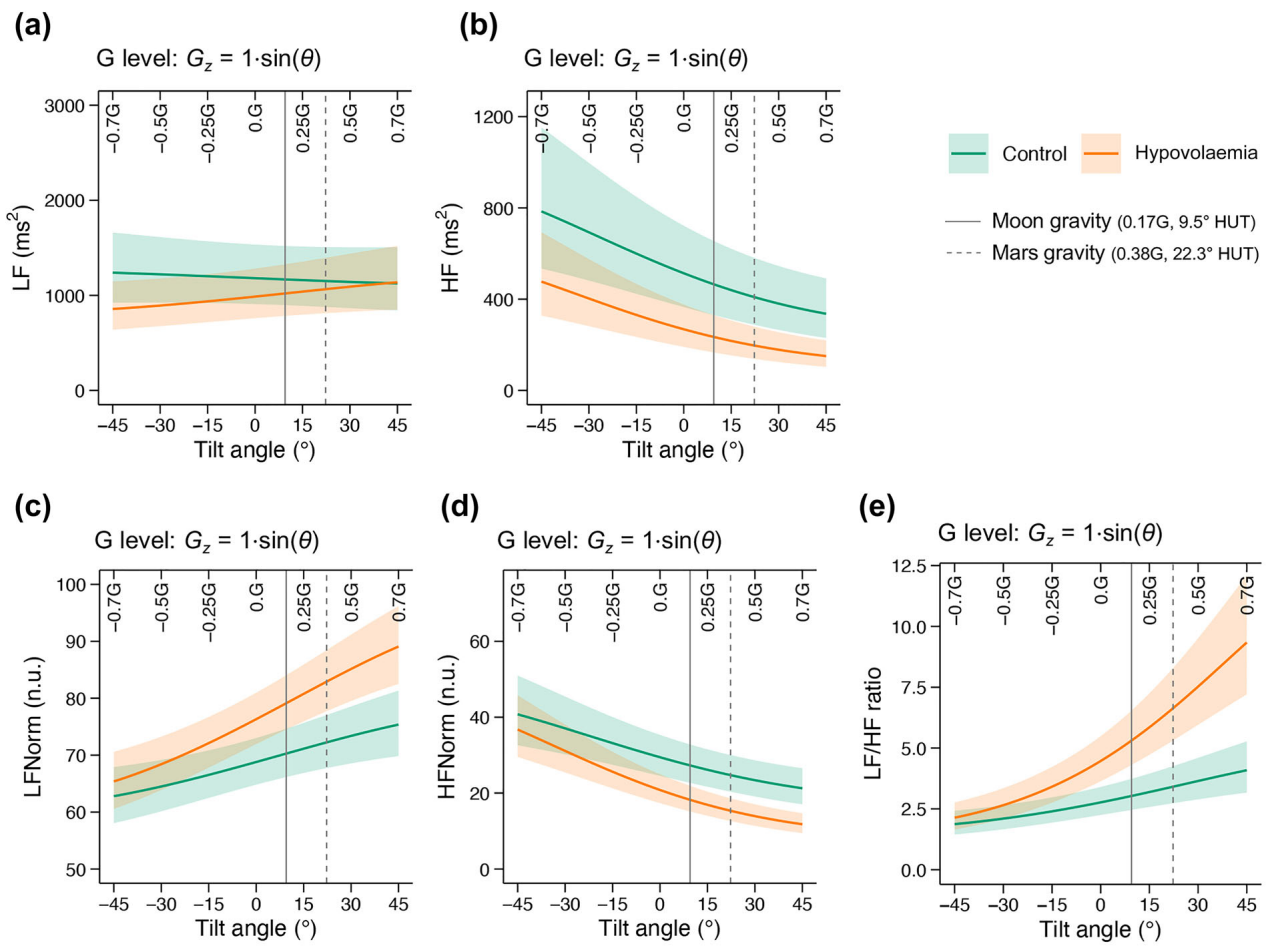
Estimated gravitational dose–response curves for frequency‐domain autonomic indices as a function of tilt angle. (a) LF, power density in the low frequency range (0.04–0.15 Hz); (b) HF, power density in the high frequency range (0.15–0.4 Hz); (c) LFNorm, LF in normalized units (n.u.); (d) HFNorm, HF in normalized units (n.u.); (e) LF/HF ratio, ratio of low to high power densities. Curves were fitted from experimental data with LMMs models as described in the main text. Curves are presented as means ± 95% confidence interval. The gravitational vector is projected along the cranio‐caudal axis for tilt as: *G* level: *G_z_* ​ = 1 × sin(θ). The vertical lines indicate the approximate partial gravity levels of the Moon (continuous line) and Mars (dashed line).

The model‐estimated gravitational dose–response curves for all haemodynamic and autonomic outcomes across the −45° to +45° tilt range excluding both baselines are presented in Figures 5, 6, 7, and the estimated coefficients and results are reported in Tables 4 and 5. Tilt angle was treated as a continuous dose using the sine of the angle (sin[θ]) to reflect the cranio‐caudal component of the gravitational vector. The figures also indicate the estimated gravity levels corresponding to the Moon (0.17*G*; continuous vertical line) and Mars (0.38*G*; dashed vertical line). Curves are shown as means with 95% CI for each condition (Control vs. Hypovolaemia). In the linear dose–response models (LMM, GLMM), coefficients were interpreted as the control slope with gravitational dose, the overall hypovolaemia‐related offset, and the condition × dose interaction, indicating whether hypovolaemia altered the sensitivity of the response to gravitational loading (Table 4). When nonlinear patterns were evident, the dose–response curves were modelled using GAMMs with subject‐specific random intercepts for HR and SV, fitting the experimental data set well, explaining respectively 84% and 83.4% of the observed deviance in the data. In GAMMs, parametric terms reflected the overall condition effect, whereas smooth terms described the shape of the dose–response curve (Table 5).

**TABLE 4:** Estimated model coefficients for the gravitational dose–response curves generated by linear mixed models (LMMs) and generalized linear mixed models (GLMMs).

	Model^1^	Link^2^	Units	Estimated coefficients^3^				Subject SD^7^
				β_0_ Intercept	β_1_ sin(Angle)^4^	β_2_ Volaemia^5^	β_3_ Interaction^6^	
Haemodynamic measurements								
CO	LMM	μ = η	L min^−1^	5.40 ± 0.19	−1.10 ± 0.13	−0.97 ± 0.09	0.30 ± 0.18	0.69
MAP	LMM	μ* =* η	mmHg	96.22 ± 2.35	8.05 ± 1.66	−0.31 ± 1.13	−0.49 ± 2.33	8.5
SBP	LMM	μ* =* η	mmHg	122.29 ± 2.77	6.12 ± 1.78	−0.87 ± 1.21	−1.02 ± 2.50	10.2
DBP	LMM	μ* =* η	mmHg	75.53 ± 2.09	8.24 ± 1.69	2.02 ± 1.15	−1.79 ± 2.38	7.5
TPR	LMM	μ* =* η	mmHg min^−1^ mL^−1^	18.98 ± 0.89	6.10 ± 0.74	3.74 ± 0.50	0.18 ± 1.04	3.06
${\dot{V}}_{O_{2}}$	LMM	μ* =* η	L min^−1^	0.20 ± 0.02	−0.01 ± 0.01	−0.03 ± 0.01	0.01 ± 0.01	0.07
Time‐domain autonomic indices								
SDNN	GLMM	ln (μ) = η	ms	4.297 ± 0.069	−0.070 ± 0.042	−0.177 ± 0.029	−0.111 ± 0.060	0.256
RMSSD	GLMM	ln (μ) = η	ms	3.853 ± 0.091	−0.401 ± 0.062	−0.338 ± 0.042	−0.020 ± 0.086	0.335
HRVTi	GLMM	ln (μ) = η	—	2.714 ± 0.055	0.002 ± 0.040	−0.144 ± 0.028	−0.130 ± 0.056	0.199
BRS	LMM	μ* =* η	ms mmHg^−1^	16.325 ± 0.046	−8.55 ± 1.254	−1.743 ± 0.860	−1.106 ± 1.787	3.305
Frequency‐domain autonomic indices								
LF	GLMM	ln (μ) = η	ms^2^	7.074 ± 0.134	−0.067 ± 0.093	−0.179 ± 0.064	0.268 ± 0.131	0.487
HF	GLMM	ln (μ) = η	ms^2^	6.241 ± 0.174	−0.650 ± 0.080	−0.600 ± 0.125	−0.215 ± 0.168	0.636
LFNorm	GLMM	ln (μ) = η	—	4.231 ± 0.030	0.129 ± 0.035	0.104 ± 0.025	0.090 ± 0.050	0.1
HFNorm	GLMM	ln (μ) = η	—	3.383 ± 0.093	−0.460 ± 0.092	−0.347 ± 0.062	−0.344 ± 0.128	0.3
LF/HF	GLMM	ln (μ) = η	—	1.018 ± 0.105	0.552 ± 0.109	0.479 ± 0.081	0.491 ± 0.156	0.343

Estimated coefficients are presented as mean ± SE. ^1^All models use a linear predictor of the form: η*_ij_ *= β_0_ + β_1_ sin(*Angle*) + β_2_ (*Volaemia_j_*) + β*_3_* (*Interaction*) + γ*_i_* + ε*_ij_* for subjects *i* (*i *= 1: 16), and *Volaemia j* (*j* = 0: 1). All GLMMs have a gamma distribution. ^2^Link function between the linear predictor, η, and the expectation of the dependent variable, μ.

^3^For GLMMs, coefficients β are given on the scale of the linear predictor for subject *i*, η*_i_* = Xβ + γ*_i_*. ^4^Sine of tilt angle from 0.707 (sin (45°)) to −0.707 (sin (−45°)), negative angles represent head‐down tilt, positive angles represent head‐up tilt. ^5^ *Volaemia_j_*: control = 0, hypovolaemia = 1. ^6^Additional coefficients for interaction effects as noted. ^7^Standard deviation, σ, of random intercept, γ, for subject *i*. $\gamma_{i}∼N\left(0,\sigma^{2}\right)$. Units for σ are the same as the estimated coefficients.

**TABLE 5:** Details of generalized additive mixed models (GAMM) analyses for two dependent variables: heart rate (HR) and stroke volume (SV).

	Parametric terms				Smooth terms^3^			Subject SD^6^	Deviance explained^7^
	Intervention^1^				sin(Angle)^4^				
	*t*	*P*	χ^2^ test for smooth^2^	Curve	EDF^5^	*F*	*P*	σ	%
HR (bpm)	11.651	< 0.001	*P* < 0.001	Control	3.26	24.409	< 0.001	6.29	84.0
				Hypovolaemia	3.37	68.801	< 0.001		
SV (mL)	−13.620	< 0.001	*P* = 0.261	—	3.02	46.266	< 0.001	17.89	83.4

Significance of parametric and smoothed terms, effective degrees of freedom of smoothers, size of subject random effect, and model goodness of fit (deviance explained). See text for abbreviations and model details. ^1^Intervention: Control or Hypovolaemia; 𝑡 reports effect size of control compared to hypovolaemia. ^2^Likelihood ratio test (χ^2^ test on the REML‐fitted models) to compare nested GAMM. Intervention‐specific smooth was performed if *P* < 0.05, otherwise a common smooth was used. ^3^Shrinkage‐penalized cubic regression splines fit to Intervention. ^4^Sine of the tilt Angle in radians, positive values indicate head‐up tilt (HUT); 𝐹 reports effect size. ^5^Effective degrees of freedom. ^6^Random effect. ^7^Goodness of fit, equivalent to the unadjusted 𝑅^2^.

## 4 DISCUSSION

While cardiovascular and autonomic responses to acute tilt have been reported previously (Hainsworth & Al‐Shamma, 1988; Nagaya et al., 1995; Porta et al., 2007; Vijayalakshmi & Madanmohan, 2006; Yamazaki et al., 2001; Zaidi et al., 2000), studies combining both directions of tilt within a single experimental framework remain limited (Robin et al., 2026; van Lieshout et al., 2005; Whittle et al., 2022), and such dose–response relationships have not been defined under hypovolaemic conditions. This study examines acute haemodynamic and autonomic responses to graded changes in body orientation relative to the gravity vector, before and during drug‐induced hypovolaemia. We generate continuous gravitational dose–response curves spanning both head‐down and head‐up tilt. Viewing gravitational loading as a continuous stimulus provides an integrative framework for describing cardiovascular regulation across intermediate loading levels, including Moon‐ and Mars‐equivalent gravity, with relevance to both spaceflight and terrestrial physiology.

### 4.1 Hypovolaemia in altered gravity

PV changes are a consistent feature of altered‐gravity exposure, particularly in true or simulated microgravity where the hydrostatic gradient is removed. Following an early thoraco‐cephalic fluid shift and neurohumoral adjustments, microgravity induces a rapid hypovolaemia (−16% in PV on Day 2) (Leach et al., 1996), which likely contributes to haemostatic and endothelial changes and to impaired orthostatic tolerance upon return to 1*G* (Blomqvist et al., 1994; Coupé et al., 2011; Gharib & Hughson, 1992; Jordan et al., 2022). Comparable PV losses are also reported during ground‐based analogues such as dry immersion (−11% on Day 2) (Robin et al., 2020, 2023) and head‐down bedrest (−13% on Day 13) (Amirova et al., 2020). In the present study, furosemide diuretic produced a ∼15% PV reduction within 3 h, consistent with other furosemide studies (Convertino & Baumgartner, 1997; Evans et al., 2015; Fu et al., 2005; Iwasaki et al., 2000; Ogawa et al., 2009, 2013; Romero et al., 2011) and closely matching the magnitude of early microgravity‐associated hypovolaemia. Together, these findings support our protocol as a controlled model of acute ‘early‐phase’ hypovolaemia superimposed on graded gravitational stress.

Beyond spaceflight and ground‐based analogues, this combination is also relevant to terrestrial medicine, where dehydration, diuresis, prolonged bed rest or acute blood loss may reduce central blood volume and contribute to orthostatic intolerance during mobilization or return to upright posture (Barbic et al., 2019a; Hristovska et al., 2023a; Schell et al., 2021; Wahba et al., 2022).

### 4.2 Central haemodynamics and autonomic response to hypovolaemia during gravitational stress

Early hypovolaemia is considered a key contributor to cardiovascular deconditioning during altered‐gravity exposure and is linked to postflight orthostatic intolerance and reduced exercise capacity (Convertino, 2009). In our study, acute hypovolaemia increased HR while SV and CO decreased, whereas TPR increased and arterial pressure remained largely unchanged. This pattern is consistent with arterial baroreflex‐mediated compensation, in which tachycardia and vasoconstriction compensate the reduction in central volume and CO to preserve blood pressure. Potassium was administered as a single prophylactic oral dose, and participants were normotensive at baseline; we thus consider it unlikely to have significantly influenced blood pressure changes. Similar haemodynamic features are reported after spaceflight during orthostatic challenge, including tachycardia (Sigaudo‐Roussel et al., 2002) and increased TPR with reduced CO and SV compared with preflight responses (Buckey et al., 1996; Levine et al., 2002). In previous studies, resting ${\dot{V}}_{O_{2}}$ changed little with tilt or gravitational changes, suggesting that pulmonary responses are not strongly driven by the *G_z_* component across these postures (Diaz‐Artiles, Navarro Tichell et al., 2019; Prisk et al., 1993; Whittle et al., 2022). In the present study, the lower ${\dot{V}}_{O_{2}}$ observed during hypovolaemia likely reflects the reduction in cardiovascular–metabolic state accompanying the fall in CO, rather than a primary effect of tilt itself.

In our study, HRV indices supported reduced vagal modulation during hypovolaemia: time‐domain metrics (SDNN, RMSSD, HRV triangular index) were lower at seated baseline and across tilt. In addition, the progressive reduction in BRS with increasing HUT is consistent with prior studies showing reduced cardiac BRS during orthostatic stress, while the lower seated‐baseline BRS during hypovolaemia suggests that cardiovagal buffering was already attenuated before the graded tilt stimulus. This is also in line with head‐down bedrest and hypovolaemia studies (Barbic et al., 2019b; Iwasaki et al., 2004) indicating that impaired baroreflex‐mediated HR regulation contributes to reduced orthostatic tolerance after cardiovascular deconditioning. Clinically, such a pattern may also be relevant in situations of dehydration, diuretic use, blood loss or early mobilization, where reduced central blood volume and impaired BRS can increase susceptibility to presyncope or syncope. Comparable reductions in time‐domain HRV and shifts toward lower HF power have been reported during mild haemorrhage induced by 450 mL blood donation, consistent with vagal withdrawal during acute central hypovolaemia (Hristovska et al., 2023b; Yadav et al., 2017).

During hypovolaemia, we found a reduction in absolute HF power and HFNorm, together with an increase in LFNorm and LF/HF ratio, consistent with reduced PV after blood donation (Yadav et al., 2017). In altered‐gravity analogues, dry immersion exposure has also been associated with a larger HF reduction and greater LF/HF increase during head‐up tilt after exposure (Miwa et al., 1997; Navasiolava et al., 2011), consistent with a reduced parasympathetic contribution to reflex control. Because LF/HF ratio is not a direct specific measure of sympathovagal balance (Billman, 2013), we interpret the frequency‐domain changes primarily as decreased parasympathetic modulation with a relative sympathetic dominance during combined hypovolaemia and orthostatic stress.

In our study, the control tilt test was always performed first and not at the same time of day as the post‐furosemide tilt test (with the 3‐h delay for furosemide effect to allow diuresis). Cardiovascular response reproducibility of HUT has been shown to be prone to important intra‐individual variations (Sagristà‐Sauleda et al., 2002), and another study found a 90% results duplicability during tilt (Grubb et al., 1992). The second session combined a marked hypovolaemic stimulus with an acute gravitational challenge, and the physiological changes observed during the post‐furosemide session (e.g., higher HR, greater sympathetic predominance) do not suggest a simple attenuation of responses that might be expected with habituation alone. Although it supports the interpretation of the observed differences being primarily driven by the hypovolaemic state, we cannot eliminate the possibility of a small contribution of protocol‐related variability, including possible time‐of‐day and sequence effects when interpreting these cardiovascular and autonomic data.

### 4.3 Countermeasure design and translational Earth implications

Our dose–response curves across tilt angles provide an integrated framework to map cardiovascular haemodynamic and autonomic responses to the gravitational component aligned with the body axis (*G_z_*), which varies predictably with tilt angle. This enables estimation of responses at partial‐gravity‐relevant levels (e.g., Moon ∼0.17*G* and Mars ∼0.38*G*, corresponding to ∼9° and ∼22° HUT, respectively), while also encompassing standard ground analogue postures such as −6° HDT used to reproduce microgravity headward fluid shift features. A key strength of this approach is that tilt is modelled as a continuous gravitational stimulus rather than as isolated postures, allowing estimation of cardiovascular responses at intermediate loading levels relevant to the Moon and Mars. The dose–response curves therefore show not only whether hypovolaemia shifts a variable overall, but also whether it changes its responsiveness to gravitational stress. This may be useful for countermeasure design, as it helps identify when volume restoration may be sufficient and when additional support may be needed at specific partial‐gravity levels. As such, our results can inform personalization of countermeasures aimed at preserving arterial pressure and orthostatic tolerance (physical exercise regimens, fluid loading strategies or compression garment protocols) across altered‐gravity transitions during hypovolaemia.

Commercial human spaceflight is expanding and includes non‐career participants, highlighting the practical importance of understanding how hydration/volume status may interact with gravitational stress (Jones et al., 2024). In general population data, 50% of adults do not meet adequate intake targets for water from fluids (according to the European Food Safety Agency criteria) (Ferreira‐Pêgo et al., 2015), and reduced thirst/fluid intake has also been described during spaceflight. In our study, the induced body water loss (∼1.5 L) can be classified as mild dehydration in adults (Dmitrieva et al., 2024), supporting the relevance of these dose–response relationships to contexts where volume depletion and postural challenge co‐occur. Beyond spaceflight, this includes rehabilitation after prolonged bed rest (Levine et al., 1997), clinical practice related to orthostatic intolerance and syncope (Bryarly et al., 2019) and unusual or rapidly changing postures (e.g., surgery in Trendelenburg position) (Likhvantsev et al., 2025). Thus, in hospital settings, these results can provide physiological support for nursing‐driven strategies such as graded repositioning, symptom‐guided monitoring, and selective use of volume repletion and external compression to reduce the risk of syncope.

### 4.4 Limitations

Several limitations should be acknowledged.

First, the control and post‐furosemide sessions were not time‐matched or randomized. The 3‐h delay on Day 2 was required to allow diuresis and a measurable PV reduction before tilt testing, whereas the control session was always performed first and in the morning. Thus, time‐of‐day effects, habituation, day‐to‐day variability, and the lack of formal standardization of sleep and dietary habits cannot be excluded. However, the clear evidence of acute volume depletion (∼15% reduction in PV and body water) supports hypovolaemia as the primary driver of the observed cardiovascular differences.

Second, the present acute graded tilt does not reproduce the full physiology of spaceflight as it does not capture sustained tissue weight removal (Buckey et al., 2018), chronic cephalad fluid shift and PV reduction occurring in the first days of flight (Watenpaugh, 2001), or the progressive neurohumoral, vascular and cardiac adaptations that develop over days to weeks (Shibata et al., 2023).

Third, endocrine mediators of volume regulation and electrolyte balance, including the renin–angiotensin–aldosterone system and vasopressin, are altered during spaceflight (Olde Engberink et al., 2023). In our study, hormonal fluid regulation was not measured, limiting mechanistic interpretation. Thus, the present study does not permit differentiation between neural reflex adjustments and humoral compensation, nor does it enable evaluation of inter‐individual variability in endocrine responses. However, the haemodynamics and autonomic data captured the integrated physiological response central to our aims. Accordingly, the present dose–response relationships should be interpreted as acute responses to graded gravitational loading during experimentally induced hypovolaemia. Finally, the relatively homogeneous sample of young, healthy and recreationally active adults limits generalizability to older adults, individuals with cardiovascular or autonomic dysfunction, or patients with impaired volume regulation or orthostatic intolerance, in whom compensatory reserve is often reduced. However, our sample of participants reduced physiological variability and improved identification of dose–response patterns.

### 4.5 Conclusion

In conclusion, acute hypovolaemia amplified cardiovascular and autonomic strain across graded altered gravity, primarily through higher heart rate and peripheral resistance, lower SV and CO, and reduced autonomic variability. Treating tilt as a continuous gravitational stimulus extends these findings beyond isolated posture comparisons and provides a quantitative framework across altered‐gravity‐relevant levels such as the Moon and Mars, and upright orthostatic stress. This approach may support future countermeasure development for gravitational transitions in spaceflight while also informing Earth‐based conditions in which altered volume status and orthostatic stress coexist.

## AUTHOR CONTRIBUTIONS

Adrien Robin, David C. Zawieja and Ana Diaz‐Artiles contributed to the study conception and design. Material preparation, data collection, analysis, and interpretation were performed by Adrien Robin, Cort D. Reinarz, Huc Pentinat‐Llurba, Syeda Yasmin Zaman, Christopher J. Rasmussen and Ana Diaz‐Artiles Medical supervision and nursing support were provided by Jason R. McKnight and Lisa Haddad. The first draft of the manuscript was written by Adrien Robin and all authors commented on previous versions of the manuscript. All authors have read and approved the final version of this manuscript and agree to be accountable for all aspects of the work in ensuring that questions related to the accuracy or integrity of any part of the work are appropriately investigated and resolved. All persons designated as authors qualify for authorship, and all those who qualify for authorship are listed.

## CONFLICT OF INTEREST

The authors have no relevant financial or non‐financial interests to disclose.

## Data Availability

The datasets analysed for this study are publicly available, a repository can be found on GitHub: https://github.com/BHP-Lab/TRISH_Furosemide_Hemodynamics.git
